# Adult Vaccination Strategies for the Control of Pertussis in the United States: An Economic Evaluation Including the Dynamic Population Effects

**DOI:** 10.1371/journal.pone.0006284

**Published:** 2009-07-16

**Authors:** Laurent Coudeville, Annelies Van Rie, Denis Getsios, J. Jaime Caro, Pascal Crépey, Van Hung Nguyen

**Affiliations:** 1 sanofi pasteur, Lyon, France; 2 Department of Epidemiology, University of North Carolina at Chapel Hill, Chapel Hill, North Carolina, United States of America; 3 United BioSource, Concord, Massachusetts, United States of America; 4 McGill University, Montreal, Canada; CIET, Canada

## Abstract

**Background:**

Prior economic evaluations of adult and adolescent vaccination strategies against pertussis have reached disparate conclusions. Using static approaches only, previous studies failed to analytically include the indirect benefits derived from herd immunity as well as the impact of vaccination on the evolution of disease incidence over time.

**Methods:**

We assessed the impact of different pertussis vaccination strategies using a dynamic compartmental model able to consider pertussis transmission. We then combined the results with economic data to estimate the relative cost-effectiveness of pertussis immunization strategies for adolescents and adults in the US. The analysis compares combinations of programs targeting adolescents, parents of newborns (i.e. cocoon strategy), or adults of various ages.

**Results:**

In the absence of adolescent or adult vaccination, pertussis incidence among adults is predicted to more than double in 20 years. Implementing an adult program in addition to childhood and adolescent vaccination either based on 1) a cocoon strategy and a single booster dose or 2) a decennial routine vaccination would maintain a low level of pertussis incidence in the long run for all age groups (respectively 30 and 20 cases per 100,000 person years). These strategies would also result in significant reductions of pertussis costs (between −77% and −80% including additional vaccination costs). The cocoon strategy complemented by a single booster dose is the most cost-effective one, whereas the decennial adult vaccination is slightly more effective in the long run.

**Conclusions:**

By providing a high level of disease control, the implementation of an adult vaccination program against pertussis appears to be highly cost-effective and often cost-saving.

## Introduction

Pertussis vaccines were first licensed in the United States (US) in 1914 and were combined with diphtheria and tetanus toxoids in 1918 [1]. Their widespread use in the 1940s for infant vaccination led to a dramatic reduction in pertussis (whooping cough) cases. In 1976, the number of reported cases in the US hit an all-time low of just over 1,000 [2]. The incidence of reported pertussis has thereafter steadily increased over the past two decades, with an increasing proportion of cases reported among adolescents and adults [3]–[6]; this resurgence means that pertussis is again a public health concern. In 2004, the Centres for Disease Control and Prevention (CDC) reported 25,827 cases of pertussis, representing an incidence of 8.9 per 100,000 US population [5]. Due to a lack of clinical awareness, insensitivity of culture and polymerase-chain-reaction (PCR) techniques, and a lack of standardized serological testing and criteria, the reported incidence is estimated to represent only a fraction of the true incidence [7]–[9]. Furthermore, under-diagnosis may occur differentially according to age, with most adult cases not being detected or reported. This is illustrated by data from the Acellular Pertussis Vaccine Trial (APERT), which reported an incidence of 370 to 450 cases per 100,000 person years in adolescents and adults [10], compared to the reported US incidence of 1.1 and 7.7 per 100,000 adults (aged ≥20 years) and adolescents (aged 10 to 19 years), respectively [5]. In addition, the APERT study found that there may be approximately five cases of asymptomatic pertussis infection for every case of symptomatic infection in adults and adolescents, which may contribute to the circulation of *Bordetella pertussis* in the community [11]. These data suggests that there may be as many as 700,000 to 8,000,000 cases of pertussis, either symptomatic or asymptomatic, in any given year in the adult and adolescent population as a whole.

In view of the clear burden of pertussis among older age groups, and to reduce transmission of *B. pertussis* in health care settings and to infants too young to be vaccinated [12]–[14], the US Advisory Committee on Immunization Practices (ACIP) has issued recommendations for routine adolescent booster vaccination [15], and routine or targeted vaccination of adults [16]. In addition, the ACIP has recently reaffirmed its recommendation for the use of TdaP in post-partum women to help to protect the newborn [17].

Determining the optimal adult vaccination strategy requires an assessment of both the economic and epidemiologic impact of alternative strategies. A recent review discussed an economic analysis of adolescent and adult pertussis vaccination [18]. Among the US-specific studies, no consensus has been reached on their economic value since different studies led to inconsistent results [19]–[22]. One cost-benefit analysis has shown that routine vaccination in both adolescents and adults provides significant health benefits and are cost savings [22] while another economic analysis concludes that routine pertussis vaccination is likely to be cost-effective for adolescents but not for adults [20]. More recently, an economic evaluation concludes that adult pertussis vaccination is cost-effective if the disease incidence in adults is greater than 120 cases per 100,000 population [21]. Despite these discrepancies, all these studies have consistently shown that the level of incidence and the potential herd immunity effects are key drivers of the results 19, 22, 23. Paradoxically, all these studies fail to properly consider these two factors. Firstly, communicable diseases are more appropriately modeled using dynamic models that are able to directly take into account the transmission of the disease in the population [24]. None of the previous studies used that approach; at best they used a potential reduction of incidence for unvaccinated individuals in their sensitivity analyses. Second, it can be hypothesized that the recent resurgence of pertussis is symptomatic of a waning of the population immunity coupled with particular transmission dynamics between age groups. Thus, a recent analysis [25], published by some of the authors of the present study, uses an epidemiological model initially developed by Hethcote [26] to analyze the potential impact of adult vaccination in the US. In particular, this study shows how the progressive shift from “naturally acquired immunity” to “vaccine-acquired immunity” triggers large fluctuations in disease incidence for some vaccination strategies; but the economic consequences of such fluctuations were overlooked.

In the present study, we aim to fill that gap by combining the results of this epidemiological model with cost data to estimate the relative cost-effectiveness of new adult vaccination strategies.

## Methods

### Epidemiological model

The cost-effectiveness analysis builds upon on a compartmental age-structured transmission model of pertussis in the US [25], which is an update of previous published transmission models of pertussis [26]–[28]. In brief, the model stratifies the US population in 50 age groups and compartmentalizes them into three main pertussis states: fully susceptible, fully or partially immune, and infectious. The infectious state is split into typical, mild and asymptomatic, each having its own duration and level of infectiousness. There are four vaccination compartments depending on the number of doses received. A difference is made between vaccine-acquired and natural immunity, both of which wane over time in three different phases (Table 1). The median duration of residence in each phase is 4 years for vaccine immunity waning and 8 years for natural immunity waning. Transfers between states occur because of vaccination and exposure to *B. pertussis*. The distribution of outcomes in the event of exposure depending on immunological status is given in Table 1. The rates of outcome do not differ from the distribution published by *Coudeville et al.* [25]. Rates of exposure (or infection) are “dynamic” and determined by the proportion of individuals in the various states and the related forces of infection.

**Table 1:** Distribution of outcomes in case of exposure.

	Type of infection					
Compartments	Typical		Mild		Asymptomatic	
Susceptible	73%	(73–73)	25%	(25–25)	2%	(2–2)
Immune	0%	(0–0)	0%	(0–0)	0%	(0–0)
Natural waning 1	10%	(10–10)	30%	(41–41)	45%	(45–45)
Natural waning 2	5%	(5–5)	15%	(15–15)	35%	(35–35)
Natural waning 3	0%	(0–0)	10%	(10–10)	30%	(30–30)
Vaccine 1	7%	(0–73)	38%	(2–25)	34%	(12–2)
Vaccine 2	1%	(0–23)	14%	(2–51)	30%	(12–20)
Vaccine 3	0%	(0–2)	7%	(2–22)	23%	(12–34)
Vaccine 4	0%	(0–0)	2%	(0–11)	12%	(4–27)
Vaccine waning 1	49%	(9–73)	42%	(42–25)	8%	(31–2)
Vaccine waning 2	5%	(1–34)	34%	(15–49)	35%	(31–14)
Vaccine waning 3	0%	(0–2)	7%	(2–22)	23%	(12–34)

Distribution of outcomes in case of contact with an infectious person according to immunological status. Values for susceptible, immune and natural waning are taken from *Van Rie et al.* [25]. Values for vaccine-related compartments are estimated using *Bisgard et al.* [*31*] and *Ward et al.* [10]. Figures in parentheses define the range used in the sensitivity analysis.

Age-specific forces of infection for symptomatic disease were calculated during the calibration process to match the observed incidence. For adolescents and adults, the force of infection was based on incidence data reported in APERT [10], [22]. For children, CDC surveillance data [5] were adjusted for under-reporting using age-specific under-reporting estimates [7]. To address the potential infectiousness of asymptomatic disease, data from a recent study suggesting that 16% of infections in infants are the result of transmission of asymptomatic disease were used to calibrate the model [29]. The age-specific forces of infection were identified using a ‘who acquires infection from whom’ (WAIFW) matrix whose structure had been used in previous publications [25], [27], [30].

Estimates for efficacy of the primary series vaccination in children were based on a recent case-control study of pertussis vaccination in children aged 6 to 59 months in the US [31]. Depending on the number of doses administered (one to four), the initial protection is estimated to range from 46.0% to 96.4%. For adolescents and adults, we used the 92% vaccine efficacy observed in the APERT trial [10].

Finally, sources of infection for infants were calculated from published data [29], [32] and are presented in Table 2, along with other key parameters used in the epidemiological model.

**Table 2:** Key parameters in the epidemiological model.

Parameter	Estimate [Range in Sensitivity Analyses]
**Duration of infectiousness** ^[**23**]^	
Typical case	4 weeks
Mild case	3 weeks
Asymptomatic case	1 week
**Vaccine efficacy** [**^10^**], [26]	
After the first dose	46.0% [0.0–88.2]
After the second dose	79.6% [24.6–94.5]
After the third dose	91.7% [74.5–97.3]
After the fourth dose	96.4% [86.4–99.0]
After booster dose	92.0% [32.0–99.0]
**Vaccine coverage**	
Childhood	Between 80% & 96% depending on age
Adolescent	75%
Cocoon strategy	65% [35%–95%]
Routine adult vaccination	40% [20%–60%]
**% of cases resulting from exposure to household contacts** ^[**27]**, [28****]^	
0–1 months	48.1% [38.0, 58.2]
2–3 months	48.1% [38.0, 58.2]
4–5 months	48.1% [38.0, 58.2]
6–12 months	33.6% [25.6–41.6]
13–18 months	39.2% [25.5, 52.9]
19–24 months	41.7% [25.0, 58.3]
2 years	38.2% [23.5, 55.9]
3 years	41.5% [26.8, 56.1]
4 years	40.5% [26.2, 54.8]
5 years	23.6% [12.7, 34.5]
**Case fatality rates** ^[**29**]^	
*Typical cases*	
<1 year	0.69% [0.52–0.86]
1–9 years	0.05% [0.04–0.06]
10–17 years	0% [0–0]
18+ years	0.03% [0.02–0.04]
*Mild cases*	
<1 year	0.0% [0–0]
1–9 years	0.0% [0–0]
10–17 years	0.0% [0–0]
18+ years	0.0% [0–0]
**Rates of long term sequelae** ^[**29**]^	
*Typical cases*	
<1 year	0.06% [0.05–0.08]
1–9 years	0.02% [0.01–0.02]
10–17 years	0.02% [0.01–0.02]
18+ years	0.02% [0.01–0.02]
*Mild cases*	
<1 year	0.0% [0–0]
1–9 years	0.0% [0–0]
10–17 years	0.0% [0–0]
18+ years	0.0% [0–0]

### Vaccination strategies

Health economic outcomes were evaluated for the following vaccination scenarios: (1) primary series vaccination in children (*Childhood vaccination*); (2) primary childhood series vaccination plus routine adolescent vaccination at 12 years of age (*childhood+adolescent*); (3) primary childhood series vaccination, routine adolescent vaccination, plus targeted vaccination of parents of newborns (i.e., cocoon vaccination) starting at 20 years of age (*Childhood+adolescent+cocoon*); (4) primary childhood series vaccination, routine adolescent vaccination, targeted vaccination of parents of newborns, plus a single dose of vaccine for adults as per the ACIP recommendation of December 2006 (*Childhood+adolescent+1 dose at 40 years*); and (5) primary childhood series vaccination, routine adolescent vaccination, plus routine vaccination of adults every 10 years *(childhood+adolescent+routine adult)*. The base case analyses assumed 80% to 96% coverage depending on age in infants and children, 75% in adolescents, 40% with routine adult vaccination, and 65% in parents of newborns [25]. We varied the coverage rate of the two latter strategies in sensitivity analysis using respectively the ranges [20%–60%] and [35%–95%].

### Cost Inputs

For children and adolescents, CDC surveillance data corrected for under-reporting were used to estimate the incidence of pertussis cases requiring medical attention. We assumed that all symptomatic infections in children and adolescents led to some medical care. For adults (20 years and above), the gap between the estimates of the incidence derived from the APERT trial and CDC surveillance corrected for under-reporting [25] led to an estimate that only 48.7% of typical cases and 0% of mild cases receive some form of medical care. Using this approach we estimated that the incidence of adult cases requiring medical attention was 90 per 100,000 individuals in 2004 (ranging in sensitivity analyses from 44 to 165).

For cases requiring medical care, age-specific costs for mild and typical pertussis cases were calculated based on published estimates [20], [33], [34] updated to 2006 levels using the US price index for medical care (available at www.bls.gov). Estimates include both direct and indirect costs, and are categorized into short-term costs and costs resulting from long-term disability or pertussis-related death (Table 3). Short-term direct costs stand for medical costs for mild or severe cough and/or pneumonia including physician visits, emergency room visits, hospitalization, chest radiographies, laboratory tests, and use of antibiotics and other drugs. Those outcomes are weighted by their rate of occurrence in the different age groups (i.e. <1 year, 1–9 years, 10–17 years, ≥18 years). Long-term direct medical care costs include the medical costs for disabilities and apply only to patients with long-term sequelae following infection. Short-term indirect costs include the costs for reduced work productivity and absence from work occurring during the pertussis episode. Time lost from non-work activities was not included. In addition we took into account the long-term indirect costs related to lost productivity from death and permanent disabilities. We assumed that mild cases never result in severe outcomes (i.e. death, permanent disability, or hospitalization).

**Table 3:** Estimates for the short term cost per case of pertussis infection (ranges used in sensitivity analyzes presented in parentheses).

	<1 year+	1–9 years+	10–17 years#	18+ years#
**Typical cases**				
Short term direct costs	$7,006 [5254–8757]	$646 [484–807]	$256 [192–320]	$338 [254–423]
Short term indirect costs	$390 [293–488]	$390 [293–488]	$174 [131–218]	$501 [376–626]
Medical disability costs per year*	$51,648 [38736–64560]	$51,648 [38736–64560]	$51,648 [38736–64560]	$51,648 [38736–64560]
Indirect costs due to disability or death per year**	$25,036 [18777–31295]	$25,036 [18777–31295]	$25,036 [18777–31295]	$25,036 [18777–31295]
**Mild cases**				
Short term direct costs	$377 [283–472]	$256 [192–320]	$219 [164–274]	$265 [198–331]
Short term indirect costs	$390 [293–488]	$390 [293–488]	$174 [131–218]	$501 [376–626]

* Applies only to the fraction of patients with long term sequelae following infection.

** Applies only to the fraction of patients with fatal cases of pertussis or long term sequelae following infection.

+ Caro et al. [43] updated to 2006 using CPI for medical care (www.bls.gov).

# Lee et al. [20] updated to 2006 using CPI for medical care (www.bls.gov).

Vaccination costs included in these analyses are those associated with vaccination of adolescents and adults only, as childhood vaccination is a component of all of the simulated strategies. For the cost of the vaccine itself, we applied the price difference between a combined Td vaccine and the price of a combined TdaP vaccine in the public sector ($14.13 available at http://www.cdc.gov/nip/vfc/cdc_vac_price_list.htm, March 01, 2007), as routine administration of Td vaccine is a long-standing recommendation in the US. For the same reason, we did not apply additional administration costs associated with routine vaccination of adolescent and adults. For the cocoon strategy, we assumed that an additional medical visit would be needed for fathers of newborns but not for mothers. These additional visits were valued at $30. Adverse events were assumed to lead to an additional medical consultation in 2% of vaccinees, or 0.74$ per vaccine dose administered [20].

### Analyses

Because the direct and indirect impact of the implementation of new vaccination strategies is not instantaneous, health and economic outcomes were examined over time, with the initiation date set as the beginning of 2006. Results are given at steady-state (when the full impact of vaccination has been reached) but also with a specific time horizon of 100 years. Costs are measured for a cohort of one million individuals. Net societal costs were used as the numerator, and discounted life years gained (LYG) as the denominator. All costs and outcomes are discounted at 3% per annum ranging from 0% to 5% in sensitivity analyses. Although not formally justified in the literature, we applied the widely used threshold of $100,000/LYG to consider a strategy as being cost-effective since several publicly funded interventions have been estimated around that threshold. A strategy is identified as dominating its comparator if both less expensive and more effective (i.e. under $100,000/LYG) than any other strategy, and as being dominated if both more expensive and less effective than its comparator [35].

Sensitivity analyses were performed to identify the influence on cost-effectiveness estimates of changes in key model parameters, including vaccine efficacy, vaccine coverage, sources of transmission to young infants, case fatality rates, rates of long-term sequelae and cost estimates. Ranges of values used in the sensitivity analyses are presented in Tables 2 and 3.

## Results

The epidemiological model predicts that adding a booster dose of pertussis vaccine for adolescents to the current US childhood vaccine schedule has a dramatic impact on the overall incidence of pertussis, with a reduction in the overall incidence of symptomatic pertussis from about 400 to less than 90 per 100,000 person years. These gains, however, partially fade away in the long run. As the average age of infection increases and immunity in adolescents wanes, the overall pertussis incidence oscillates and reaches a new steady-state of approximately 450 per 100,000 person years by 2060. The addition to adolescent and childhood immunization of a selective vaccination targeting the parents of newborns (i.e. cocoon strategy) would mitigate this increase and lead to a steady state of 320 cases of symptomatic pertussis per 100,000 person years. The two other adult strategies are able to achieve and maintain a low level of pertussis incidence when combined with childhood and adolescent programs: routine decennial adult immunization and the combination of the cocoon strategy with a single booster dose for all adults at 40 years of age (30 cases per 100,000 person years).

**Figure 1:**
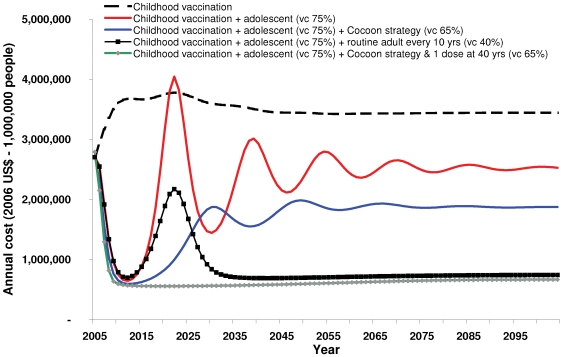
Average annual predicted costs of pertussis and vaccination from 2006 to 2106.

The impact of the different vaccination strategies on total pertussis costs over time is shown in Figure 1. The evolution of pertussis costs over time closely matches the evolution of pertussis incidence. This stems from the fact that for the different strategies considered, associated savings in pertussis treatment costs exceed additional vaccination costs. Among all new strategies, the one including the cocoon strategy and a single dose for all adults is the least expensive whatever timeframe is considered. The only other strategy that results in similar costs is the one involving decennial routine vaccination for adults. This is also illustrated by the results presented Table 4. As compared to the current childhood vaccination strategy, the savings in disease-related costs for the strategy including adolescent, cocoon and a single adult booster vaccination ($2,581,361) are 6 times the additional vaccination costs ($499,132); 3.5 times if one only considers direct treatment costs. In terms of cost-effectiveness results, the strategy including one adult booster dose clearly dominates other strategies in the base case. The only exception is the strategy including routine decennial vaccination for adults but, while not being dominated at steady-state, it is associated with a very high cost-effectiveness ratio ($678,523).

**Table 4:** Cost-effectiveness results in the base case.

	Childhood vaccination	Childhood+adolescent	Childhood+adolescent+cocoon	Childhood+adolescent+cocoon+1 dose at 40 yrs	Childhood+adolescent+routine adult
**Costs/Year–Steady state situation** $					
Vaccination Cost*	$0	$192,859	$385,840	$499,132	$623,950
Disease cost					
-treatment related	$1,184,025	$681,428	$455,238	$49,616	$37,524
-short-term indirect	$1,140,057	$814,248	$575,076	$60,012	$41,029
-sequelae-related	$611,767	$423,178	$288,640	$28,476	$20,528
-long-term indirect	$296,551	$205,133	$139,917	$13,804	$9,951
**Total costs**	$3,232,400	$2,316,846	$1,844,711	$651,040	$732,981
**Outcomes/Year** $					
Deaths	0.79	0.55	0.37	0.04	0.03
Sequelae	0.58	0.46	0.33	0.03	0.02
Life years lost	18.01	10.85	6.80	0.71	0.59
**Cost per life year gained** †					
	**Reference**	Dominating	Dominating	Dominating	Dominating
	Dominated	**Reference**	Dominating	Dominating	Dominating
	Dominated	Dominated	**Reference**	Dominating	Dominating
	Dominated	Dominated	Dominated	**Reference**	$682,842

* Cost for vaccinating adults and adolescents excluding those for doses given to children.

† Calculated by comparing each strategy to the one chosen as reference. A strategy is identified as dominating its comparator if both less expensive and more effective (i.e. under $100,000/LYG) than any other strategy, and as dominated if both more expensive and less effective than its comparator. Cost per life year gained is shown for a strategy more effective but more costly.

$ Costs and outcomes are reported for 1,000,000 people, steady state situation corresponds to a period at which the full impact of the vaccination strategy considered has been reached.

**Figure 2:**
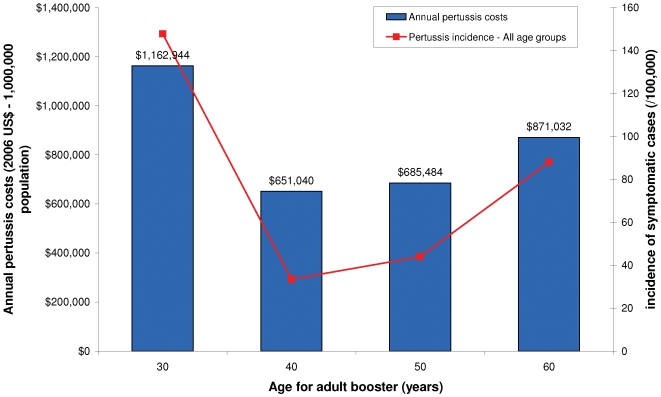
Variation in pertussis incidence and costs according to the age at which the adult booster dose is administered (Childhood vaccination+adolescent+cocoon+1 booster dose for adult vaccination - steady-state situation).

The age at which the single adult booster dose is administered plays a key role in the value of this vaccination (Figure 2). When one considers the steady-state situation (the full impact of adult vaccination strategies) 40 years of age is the optimal age for this booster dose both in terms of cost and effectiveness.

### Sensitivity Analyses

The sensitivity analyses (Table 5) demonstrate vaccine efficacy and disease incidence as the only factors to have a strong effect on the cost-effectiveness results. Neither variation of vaccination coverage, discount rate, costs, nor the extent of the cocoon effect change the dominant strategy identified in the base case.

**Table 5:** Sensitivity analyzes on cost per discounted life year gained at steady state.

	Childhood+adolescent	Childhood+adolescent+cocoon	Childhood+adolescent+cocoon+1 dose at 40 yrs	Childhood+adolescent+routine adult
**Base case**	Dominated	Dominated	**Dominating**	$678,523
**Vaccine efficacy**				
*High*	**Reference**	$128,295	$1,190,068	Dominated
*Low*	Dominated	Dominated	Dominating	**$95,962**
**Pertussis incidence**				
*High*	Dominated	Dominated	Dominated	**Dominating**
*Low*	Dominated	Dominated	**Dominating**	>$1,000,000
**Vaccination coverage**				
*High*	Dominated	Dominated	**Dominating**	>$1,000,000
*Low*	Dominated	Dominated	**Dominating**	Dominated
**Costs, case fatality & sequelae**				
*High*	Dominated	Dominated	**Dominating**	$471,812
*Low*	Dominated	Dominated	**Dominating**	>$1,000,000
**Discount rate**				
*0%*	Dominated	Dominated	**Dominating**	$482,052
*5%*	Dominated	Dominated	**Dominating**	$837,402
**Infection of infants and children via household contacts**				
*High*	Dominated	Dominated	**Dominating**	$999,924
*Low*	Dominated	Dominated	**Dominating**	$497,674

The cost per discounted life year gained is calculated by comparing each strategy with the strategy on its left. A strategy is identified as dominated if another one is both more effective and less expensive, and as dominating if it is more effective and less costly than any other strategy or is always under the $100,000/LYG threshold. In other cases, the cost effectiveness ratio is given taking as reference the dominating strategy or the one indicated as reference because none is dominating.

Optimal strategy in bold (i.e. dominating its comparator or with an ICER below $100,000/LYG).

In the low vaccine efficacy scenario - i.e. lower bound of the confidence interval derived from *Bisgard et al*.[31] and *Ward et al.* [10] - implementing routine decennial vaccination appears, with a ratio below $100,000, to be a more cost-effective solution than cocoon vaccination and a single booster dose at 40 years of age. The situation is reversed for a high vaccine efficacy scenario: implementing only the adolescent or adolescent and cocoon seems to be sufficient and the cost-effectiveness ratio is higher than $1,000,000 for the booster dose at 40 years of age.

A single booster dose at 40 years of age in addition to the cocoon strategy remains the dominant strategy in the low incidence scenario. The routine decennial vaccination for adults becomes the dominant strategy for the high incidence scenario.

## Discussion

This economic evaluation in the US is the first to build upon the results of a pertussis transmission model to assess the economic value of pertussis vaccination strategies. This kind of approach takes into account some specific features of pertussis transmission. For example, the progressive switch from naturally acquired immunity to vaccine-acquired immunity is an essential characteristic. It can trigger severe fluctuations in disease incidence which impact the cost of strategies. This argument alone demonstrates the relevance of considering disease dynamics when assessing the economic value of a vaccination program. But, more importantly, such as *Beutels et al.* [36] we do think that the capacity to prevent individuals from infecting others should not be overlooked in the assessment of the value of vaccines. This is of particular importance for pertussis for which the age group with the highest death rate (newborns) can only be protected indirectly.

Our results clearly highlight the epidemiologic and economic value of adult pertussis vaccination when combined with a routine childhood and adolescent schedule. Adult pertussis vaccination is not only cost-effective but also cost-saving in most cases.

Combining a cocoon strategy targeting parents of newborns and a single booster dose for all adults at 40 years of age appears to be the most cost-effective solution over a wide range of scenarios. Routine decennial vaccination is predicted to lead to only a slightly higher level of costs and a slightly lower incidence, and is optimal in the low vaccine efficacy or high incidence sensitivity analysis. If one goes beyond strict economic criteria for evaluating strategies and includes implementation issues, routine decennial vaccination for adults may appear as the preferred strategy due to the existence of a decennial Td program. Consequently, a simple replacement of Td by TdaP would enable the anti-pertussis strategy to capitalize on the current uptake of adult Td vaccination. As a matter of fact, US data for 2007 published by CDC show that 57.2% of 18 to 65 years old [54%–60.5%, CI 95%] had received a tetanus vaccine in the past 10 years [36]. For adolescents, more than 72% had received either Td or TdaP [70%–74%] [36] since the age of 10 years.

The fact that the age at which the adult booster dose is administered modifies the economic value of a single adult booster strategy is also worth mentioning. On this last point, it is important to keep in mind that the results presented in Figure 2 correspond to a steady-state situation (i.e. a period at which adolescent and cocoon vaccination have been implemented for long enough to reach their full impact). It is unclear whether similar findings would be true over the short term.

Three other studies have evaluated the health economic implications of adolescent and adult pertussis vaccination strategies in the US [20]–[22]. Purdy et al. performed a cost-benefit analysis and calculated the vaccine break-even cost for seven different adolescent and adult immunization strategies. They concluded that routine immunization of adolescents would be the most economical strategy. Even though routine adult vaccination every 10 years was also justified by their analysis, the authors stated that the recommendation of routine adult vaccination requires more certainty about key parameters including the duration of immunity, program costs, compliance, and non-medical costs associated with pertussis [22]. The authors did recognize the limitation of excluding herd immunity in their assessment, and acknowledged that including herd immunity may increase the benefits of immunization.

A study by *Lee at al* [20] concluded that vaccination of adolescents might be cost-effective, but that the addition of an adult vaccination component was unlikely to be economically efficient. In a subsequent analysis, more directly focused on adult immunization [21], the same authors concluded that one-time and decennial booster vaccination would be cost-effective if the disease incidence in adults was greater than 120 cases per 100,000 population.

The analytic framework used in this analysis leads to different conclusions. First, conditioning economic results to an incidence-based threshold does not appear relevant if one considers the predictions of our model. Using a threshold implicitly means that the disease incidence is expected to remain stable if the vaccination schedule is left unchanged. But our model predicts that in the absence of any adolescent or adult program, pertussis incidence will continue to increase especially amongst adults (90 per 100,000 person years in 2004, 231 in 2022). This prediction is consistent with the trend observed by CDC surveillance over recent years [3]–[5]. In this context, the main positive value of implementing an adolescent and adult program is the ability to prevent the resurgence of pertussis incidence in years to come.

The second major difference with *Lee et al.* study rests on the treatment of herd immunity. Their analysis only included herd immunity in sensitivity analyses but limited this to the effects of vaccinating adults on the incidence of pertussis in infants. The transmission model used in our analysis predicts that routine decennial vaccination for adults would not only benefit adults and infants but would also reduce pertussis incidence amongst children and adolescents by more than 90% as compared to an immunization schedule that only targets children and adolescents.

The rationale for using a dynamic epidemiologic model in these analyses is to be able to capture both the direct and indirect benefits of vaccination, as well as changes over time in the epidemiology of pertussis expected following the implementation of immunization programs. Economic evaluations conducted for other infectious diseases have highlighted the importance of including herd immunity. Recent examples include evaluations of meningococcal disease [37] and hepatitis A [38]. In the latter case, the inclusion of herd immunity effects more than doubled the predicted savings over a 10 year period.

As with all models, our pertussis model is a simplified description of the underlying processes that lead to disease and of resource utilization. Furthermore, the selection of a specific simulation technique, economic methods and parameter values is essential but results in important limitations [39]–[41].

As is often the case in economic evaluations, we had to deal with the uncertainty characterizing some of the key parameters of the analysis. This uncertainty arose due to incomplete knowledge of biological, demographic epidemiological, medical and economic factors. The true incidence of infection across age groups in the US is unknown. Estimates in the transmission model were based on calibration of the model to data on incidence and under-reporting of cases in the US, and uncertainty was addressed in sensitivity analyses.

There are also limited data available on the costs associated with mild, as opposed to typical, disease and there are no data on unreported symptomatic infections, requiring us to rely in part on assumptions (e.g., that mild disease would never lead to hospitalization), and expert opinion [42]. We took a conservative approach to assigning costs to cases of mild infection. We could alternatively have considered that mild cases in specific populations, e.g. the elderly, also require medical attention specifically for pertussis and/or as a trigger for another health event.

Mass vaccination can produce complex population effects that make the type of model selected a critical factor [23]. To reduce complexity, our model assumed a fixed vaccination schedule over a 100 year period. The influence of immigration, possible changing coverage rates over time, and potential changes in the virulence of the *B. pertussis* organism, were also not evaluated.

### Conclusions

Our simulation of the temporal evolution of pertussis epidemiology and cost-effectiveness of vaccination strategies lends support to pertussis adult vaccination strategies. In particular, our analysis supports the hypothesis that the use of a pertussis vaccine in adults in the US in addition to the childhood and adolescent program could provide considerable health benefits and could be economically viable. The vaccination strategy combining targeting parents of newborns and a single booster dose for adults at 40 years of age appears to be the most cost-effective solution. However, routine decennial vaccination would result in almost similar costs and reduction of disease incidence. This last strategy may be easier to implement since a decennial Td program is already in existence.
